# Extracranial Vertebral Artery-Internal Jugular Vein-Spinal Vein Fistula in Neurofibromatosis Type I: Case Report and Literature Review

**DOI:** 10.3389/fneur.2022.855924

**Published:** 2022-04-28

**Authors:** Jiarui Chen, Tuo Liang, Jiemei Cen, Jie Jiang, Tianyou Chen, Hao Li, Chong Liu, Jing Chen, Xinli Zhan

**Affiliations:** ^1^Department of Spine and Osteopathic Surgery, The First Affiliated Hospital of Guangxi Medical University, Nanning, China; ^2^Respiratory Medicine, The First Affiliated Hospital of Guangxi Medical University, Nanning, China; ^3^Department of Vascular Surgery, The First Affiliated Hospital of Guangxi Medical University, Nanning, China

**Keywords:** arteriovenous fistulas, neurofibromatosis type I, embolization, fistula, interventional therapy

## Abstract

**Background:**

A cervical arteriovenous fistula (AVF) in neurofibromatosis type I (NF-1) is uncommon, and it brings challenges and difficulty in treatment.

**Case Presentation:**

A 39-year-old woman was diagnosed with an NF-1-associated spontaneous vertebral artery-internal jugular vein-spinal vein fistula. The fistula was placed by coil embolization. Postoperative examination showed that the fistula closure was satisfied, and the patient's abnormal clinical manifestation disappeared without any complications after 24 months of interventional embolization. As per the literature, interventional embolization is currently the main treatment method, and it has the distinguishing features of less trauma, quick recovery, and a good prognosis.

**Conclusion:**

NF-1 associated with a spontaneous arteriovenous fistula is rare in clinical practice, which carries significant challenges in treatment, but can be effectively treated using endovascular embolism. Endovascular embolism could be the potential choice of treatment in NF-1 associated with AVF.

## Introduction

Neurofibromatosis type 1 (NF-1) is an autosomal dominant familial neurocutaneous disease, and it accounts for about 1/3,000 (1). NF-1 is also known as von Recklinghausen's disease and is caused by mutations in the tumor suppressor gene NF-1 (17q11.2) (2–4), which encodes neurofibromin that can activate cell proliferation *via* downregulating the Ras-Raf/MAPK signaling pathway (5). It is characterized by multiple café au lait macules (CALMs) on the skin, i.e., pigmented patches, multiple neurofibromas of the peripheral nerve, and optic pathway gliomas (OPGs) (6). Occasionally it is complicated by vascular diseases, such as vascular stenosis, occlusion, aneurysms, and arteriovenous fistulas, which are relatively rare (7–13).

It is well known that the incidence of congenital vascular malformations is very low, whereas the number of NF-1-related vascular abnormalities is higher. Its rate of occurrence is 0.4 to 6.4% (14–16) and is mainly found in the aorta and renal arteries (17). Patients suffering from cervical arteriovenous fistulas (AVFs) associated with NF-1 are very rare (18), and this is usually developed from trauma and medicine. The etiology of NF-1-related AVF remains unclear (18), and treatment is facing serious challenges.

Here, we reported one case of a vertebral artery-internal jugular vein-spinal vein fistula with NF-1 and gave a general view of the existing works of literature in this article.

## Case Presentation

**Figure 1:**
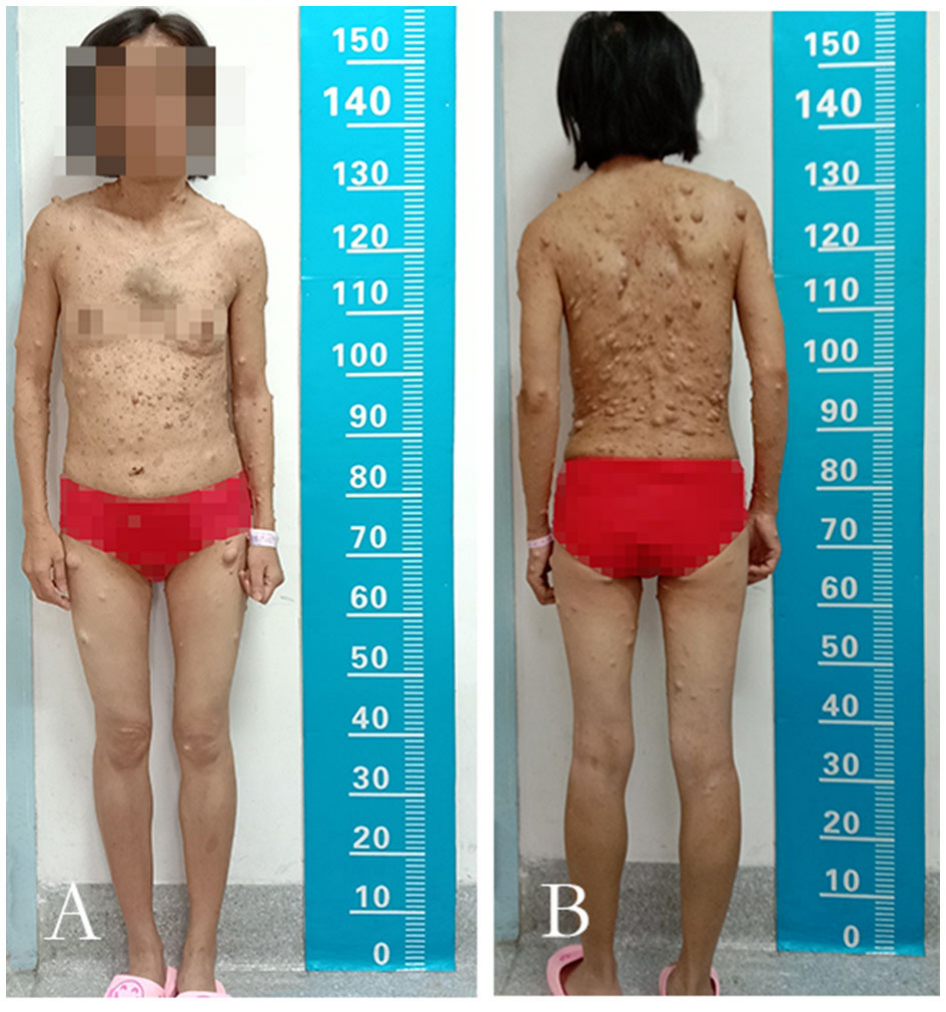
The anterior view and the posterior view of the patient. **(A,B)** The image showed innumerable masses spread throughout her body and mainly concentrated on the face, neck, bilateral upper limbs, and trunk.

**Figure 2:**
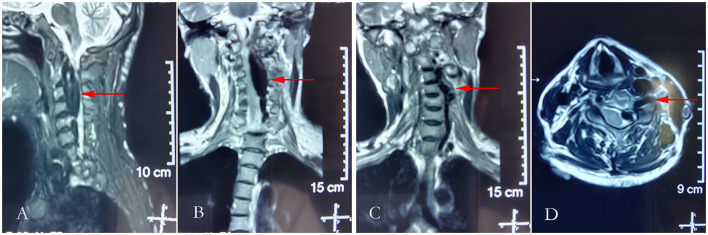
Cranial T2-weighted magnetic resonance imaging. **(A)** Sagittal plane of the neck; **(B,C)** the coronal plane of the neck; and **(D)** horizontal plane of the neck.

**Figure 3:**
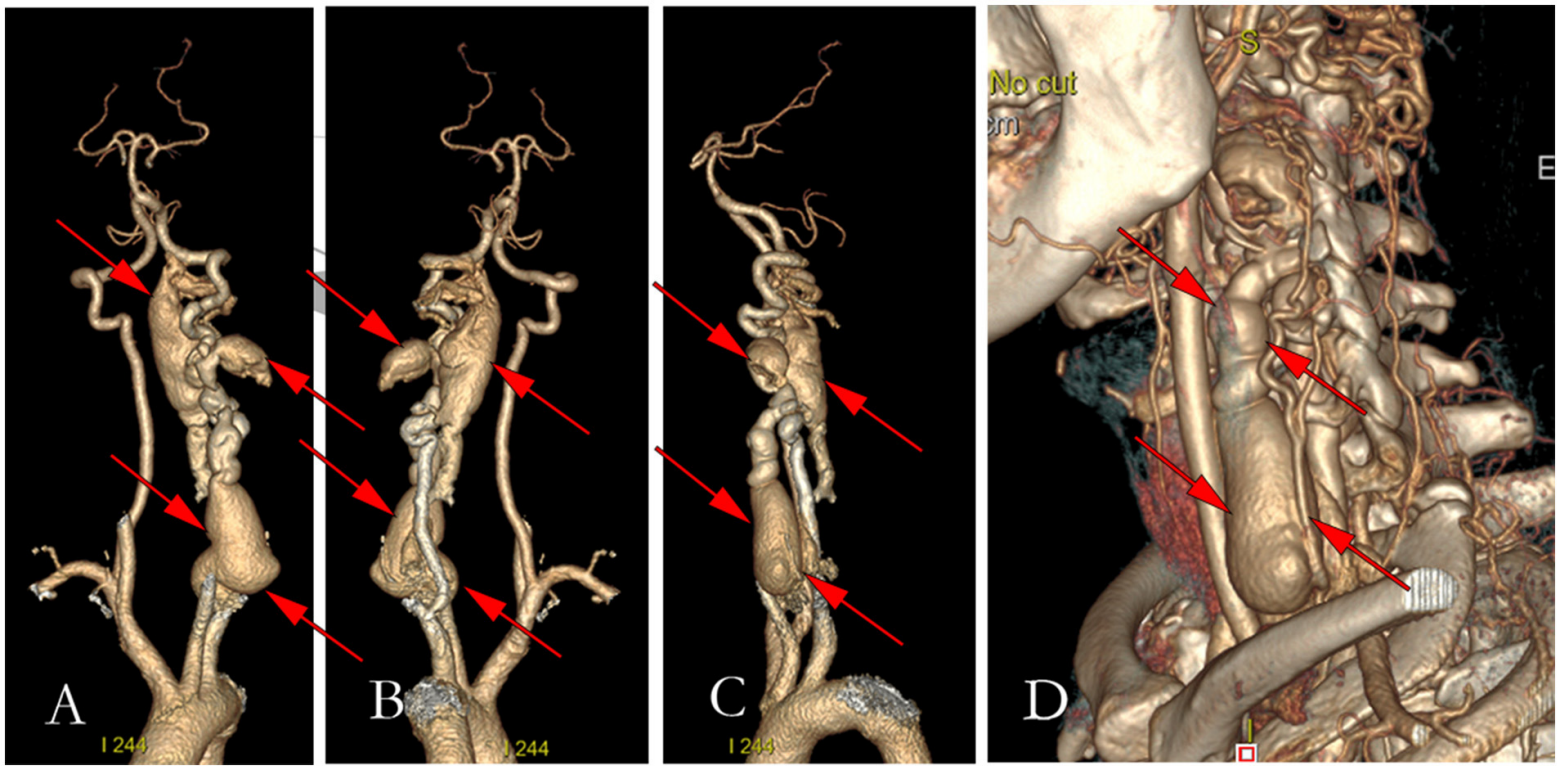
Figures of preoperative three-dimensional computerized tomography. **(A)** The anterior view of the three-dimensional CT of vertebral arteriography; **(B)** The left view of the three-dimensional CT of vertebral arteriography; **(C)** The post view of the three-dimensional CT of vertebral arteriography; **(D)** Three-dimensional reconstruction of the left neck.

**Figure 4:**
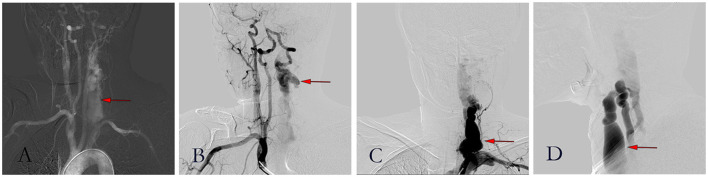
Figures of digital subtraction angiography. **(A)** Angiography showed a large area of the left vertebral artery; **(B)** Brachiocephalic trunk angiography showed a large flow of blood from the right vertebral artery through a circle of Willis to the left; **(C)** Posterior-anterior image of left vertebral arteriogram; **(D)** Lateral image of left vertebral arteriogram.

**Figure 5:**
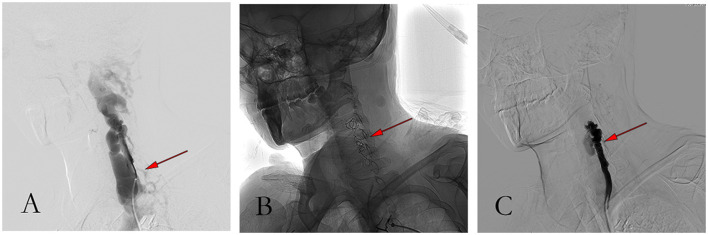
Pictures of the procedure. **(A)** Angiography showed massive venous development before embolization; **(B)** Angiographic image after coil embolization; **(C)** Angiography showed good occlusion and less vein development after coil embolization.

A 39-year-old woman with NF-1 for 32 years was admitted to our hospital with the complaint of the existence of a left cervical mass with tremors for 8 years without any inducement. Physical examination demonstrated multiple subcutaneous masses in different sizes and CALMs scattered all over the body (Figure 1). A neck mass approximately 4 × 3 cm in size was palpable on the left side of the neck, with a hard texture, poor mobility, active tremor, and continuous mechanical vascular murmur. The strength of the left limb muscle was weakened with a grade of III. Pathologic signs examination indicated the bilateral Babinski sign and positive patellar and left ankle clonus. MRI showed abnormal signals on the left side of the neck and the spinal canal of the neck. Arteriovenous malformations, neoplastic dilatation, and thrombosis were observed on the left side of the neck, also, a few blood vessels were found to protrude into the spinal canal to compress the cervical spinal cord (Figure 2). Three-dimensional CT showed an enlarged diameter of the V2 segment of the left vertebral artery that communicated with the internal jugular vein and spinal cord vein. The corresponding diameter was significantly enlarged, and the spinal cord of the C2-C5 vertebral segments was significantly compressed. It revealed the left vertebral artery-internal jugular vein-spinal vein fistula (Figure 3). Vertebral angiography showed a dilated left vertebral vein at the later stage of the artery, and hence, an arteriovenous fistula was considered (Figure 4). Before embolization, the angiogram showed multiple fistulas (Figure 5A) and three 8–12 double-plug spring coils. One 8 mm × 20 cm controllable spring coil and another 6 mm × 20 cm controllable spring coil [Axium MicroFX Coils (ev3; Plymouth, Minnesota, USA)] were applied to the main part of the fistula (Figure 5B). During the angiography, post-placement of the spring coils revealed significant occlusion, and vein development was greatly reduced compared to before (Figure 5C). After the coil embolization, 24-month follow-up demonstrated significantly improved muscle strength of the left limb with a grade of IV to V, though it was still slightly abnormal compared to the right limbs, and the abnormal numbness of the left upper limb disappeared fully.

## Discussion

NF-1 is known as an autosomal dominant familial genetic disease, which partly occurs due to genetic mutations in all ethnic groups (3). In our case report, the patient was diagnosed with neurofibromas by pathological examination in another hospital when she was young and denied any family member had the same clinical manifestation. NF-1 connected with a spontaneous arteriovenous fistula is very uncommon in clinical practice.

In the literature, there were 49 cases of AVF associated with NF-1, including our report. Moreover, spontaneous AVF takes up the majority of all cases, about 40 cases. Among the remaining cases, four cases had a history of operation, and five cases had trauma. NF-1-associated spontaneous AVF cases occurred more in women, accounting for approximately 78.38% compared to 24.32% in men, see Table 1, whereas in the two cases, gender remained unidentified. The average age of spontaneous AVF in NF-1 was 38.77 years (39.47 ± 14.02 years), with a maximum age of 70 years old (19), while the youngest suffering from the disease had just been born (20). Additionally, spontaneous AVF occurred significantly in the neck, in approximately 77.5% (31/40), which does not match with previous reports (17). However, AVF took place in the head in approximately 10% (4/40) of cases, and the chest in 5% (2/40) of cases. Along with this, spontaneous AVF occurred on the left side and right side in 58.33% (21/36) and 33.33% (12/36) of cases, respectively, while two cases had bilateral spontaneous AVF.

**Table 1:** Summary of clinical information of spontaneous arteriovenous fistula with neurofibromatosis.

															**Treatment**							
	**Gender**			**Position**					**Side**				**Aneurysm**		**Op**			**Em**			**Op and em**	**NA**
	**M**	**F**	**NA**	**H**	**N**	**T**	**H and N**	**NA**	**L**	**R**	**B**	**NA**	**Yes**	**NO**	**Re**	**Ot**	**NA**	**Re**	**De**	**NA**	**Re**	
	9	29	2	4	31	2	1	2	21	12	3	4	8	32	3	1^#^	1	25	2^*^	1	4	3
To^*^	40			40					40				40		5			28			4	3

*M, male; F, female; NA, not available; H, head; N, neck, T, thorax; Ot, other; L, left; R, right; B, bilateral; Op, operative; Em, embolization; Re, recovery; De, dead; To, total*.

# *The patient left with neck pain and post-laminectomy kyphotic deformity*.

* *One patient died from pneumonia*.

The exact pathogenesis of abnormal blood vessel appearance associated with NF-1 remains unclear. Deans (21) thought that vascular smooth muscle dysplasia results from the attenuation of intimal hyperplasia or arterial wall cell proliferation, which leads to the weakening of the arterial tube wall, aneurysm, and artery ruptures which eventually connect to the adjacent veins. Alternatively, arteriovenous malformations are due to mesoderm dysplasia. Riccardi hypothesized that the expression of neurofibromin in endothelial and smooth muscle cells of blood vessels would change due to the mutation of the NF-1 gene, and the maintenance effect of neurofibromin on blood vessels would be lost (22), eventually leading to inflexible blood vessels. In the literature, there were 80% of patients (32/40) with pure NF-1-related spontaneous AVF and 20% of patients (8/40) with both aneurysms and spontaneous AVF.

AVF with NF-1 is rare in clinical practice, though there is no difficulty in diagnosis with CT angiography (CTA) (23), magnetic resonance angiography (MRA) (24), and digital subtraction angiography (DSA). However, still, there are challenges and difficulties for good treatment. The purpose of treatment is to completely seal the fistula. Alternative treatments include traditional open surgery (ligation, reconstruction, etc.) and interventional embolization (balloons, coils, tissue glue, vinyl alcohol polymers, etc.) (25–30). However, whether it is traditional open surgery or interventional embolization, vascular fragility associated with NF-1 brings great risk to treatment, viz., rupture of blood vessels and bleeding. Compared with surgical treatment, the initial failure rate and recurrence rate were higher (31, 32). However, with the advancement of endovascular embolization materials and surgical techniques, the success rate of intravascular interventional therapy has greatly improved (31–33). In addition, interventional therapy has certain features, such as less damage, higher safety, and quick recovery after treatment (29, 34–36). Therefore, endovascular interventional therapy is undoubtedly the best choice.

Detachable balloons are best suited for fistula occlusion as they can be inflated and contracted repeatedly before separation to achieve precise fistula placement and optimal occlusion. However, if the balloon is deflated, it can result in recurrence of the fistula, and thus, it is rarely used in embolization. Particulate or liquid embolic agents are not feasible for AVF, as there are likely to be swept away by rapid flow without blocking the fistula and cause accidental embolization in other blood vessels. The detachable coil attracts negatively charged blood components (red blood cells, white blood cells, platelets, etc.) to electro-coagulate, forming a thrombus in the vessel. Due to its good circling compliance, the coil can be adjusted if the position is not satisfied and shows advantages to embolization.

In our case, if open surgery is performed, it is necessary to cut the vertebral artery foramen, lamina, etc., which is more traumatic and may affect the stability of the spine. DSA demonstrated that the fistula had a dual vascular nutrient supply, i.e., from the left vertebral artery and the right vertebral artery through to the cranial base artery ring. Occlusion *via* the retrograde approach is extremely difficult and risky, as it has to travel along the long and inward-curving vascular skull. Thus, occlusion was achieved through an antegrade approach (37) using coils. After the placement of the spring coil, the angiography showed that adequate occlusion was achieved. After 24 months of follow-up, the patient's symptoms and signs disappeared, which indicated that the treatment we chose had a favorable outcome. However, there is no standard for endovascular treatment which is recognized as excellent.

There are many methods of endovascular embolization for the treatment of AVF. Embolic agents can vary, such as balloons, coils, tissue glue, and vinyl alcohol polymers, as per the size of the fistula or different hemodynamics. Along with all the advancements and benefits of the embolization process, downside factors, such as the occurrence of new arteriovenous fistulas or rupture of blood vessels, should be considered. These secondary damages take place as the blood vessels are fragile.

## Conclusion

In clinical practice, the occurrence of NF-1 associated with spontaneous arteriovenous fistula is very uncommon. It mostly occurs in female patients and often in the left blood vessel. Literature findings illustrated that endovascular embolization treatment has noticeable benefits.

## Data Availability Statement

The raw data supporting the conclusions of this article will be made available by the authors, without undue reservation.

## Ethics Statement

Written informed consent was obtained from the individual(s) for the publication of any potentially identifiable images or data included in this article.

## Author Contributions

JiaC, JieC, and TL performed the research and wrote the paper. JiaC, JieC, TL, and JJ designed the research study. TC, HL, and CL contributed essential reagents or tools. JiaC, JieC, and CL analyzed the data. JieC, JinC, and XZ reviewed the manuscript. All authors have read and approved the manuscript.

## Conflict of Interest

The authors declare that the research was conducted in the absence of any commercial or financial relationships that could be construed as a potential conflict of interest.

## Publisher's Note

All claims expressed in this article are solely those of the authors and do not necessarily represent those of their affiliated organizations, or those of the publisher, the editors and the reviewers. Any product that may be evaluated in this article, or claim that may be made by its manufacturer, is not guaranteed or endorsed by the publisher.
