# Supplementary material for: Extracranial Vertebral Artery-Internal Jugular Vein-Spinal Vein Fistula in Neurofibromatosis Type I: Case Report and Literature Review
Source: Front Neurol. 2022 Apr 28;13:855924. doi: 10.3389/fneur.2022.855924 (PMC9104120; doi:10.3389/fneur.2022.855924)
Supplement: Supplementary file 1 [file Table_1.pdf]

Supplementary Materials: Summary of clinical information of arteriovenous fistula in neurofibromatosis type 1 patients.

Author/Year	Age (years)/ Gender	Time (mouth)	Position /Side	Symptoms	Aneurysm	Treatment	Outcome	Follow-up(month)	History
Deans(1982)[1]	45/F	3	C4-5/L	left upper extremity weakness with neck and shoulder pain	NA	Operation /embolization	Recovery	NA	NA
Deans(1982)[1]	58/F	0	head and neck/L	massive swelling over the left side of head and neck	NA	Operation /embolization	Recovery	NA	Operation
Parkinson(1986)[2]	54/F	21	C1 to foramen magnum /L	bruit	NA	Operation	Recovery	8	NA
Kubokura(1987)[3]	38/F	0	C3-5/L	left cervical pain	NA	Embolization(Balloon)	Recovery	1	NA
Tsuha(1988)[4]	31/M	0	Temporal/L	painful left temporal swelling	Yes	Embolization(Sponzel, coil)	Mild left facial paresis and painless left temporal bulging	12	trauma
Hasegawa(1989)[5]	47/M	NA	C3-5/L and R	suboccipital pain and a bruit in the right, upper cervical region	NA	Embolization and operation	Recovery	6	Operation
Wada(1989)[6]	24/F	NA	C1-6/L	neck pain and weakness in the left	NA	Operation and	Paralysis	19	Traum

				upper and lower extremities		embolization			a
Schievink(1991)[7]	28/F	4	C4/R	progressively enlarging nontender swelling	NA	Operation	Recovery	12	NA
Terada(1993)[8]	40/F	NA	Occipital	Tinnitus, vertigo, and vomiting	NA	Operation	NA	NA	NA
Cluzel(1994)[9]	48/M	6	C6/L	left C6 radiculopathy	NA	NA	NA	NA	NA
Cluzel(1994)[9]	25/F	NA	C4-6/R	paranesthesia in the right arm	NA	Embolization(Balloon)	Recovery	3	NA
Yilmaz(1997)[10]	28/M	NA	Right face	Burning and itching of the eyes	NA	Operation and embolization	Recovery	NA	Operation
Koenigsberg(1997)[11]	34/F	NA	C5-6/R	neck pain with radiculopathy to the right shoulder and arm, progressive difficulty walking, and urinary incontinence	NA	Embolization(Balloon)	Recovery	2	NA
Murayama(1999)[12]	27/F	NA	C3-5/R	right shoulder weakness and numbness, a bruit in the neck	NA	Embolization(Balloon)	Recovery	16	NA
Ushikoshi(1999)[13]	40/F	NA	C1/L	Occipitalgia, a large subcutaneous	Yes	Embolization(Coils and	Recovery	12	NA

				mass in the left suboccipital area.		polyvinyl alcohol particles)			
Ma(2000)[14]	32/F	NA	NA	subacute onset of left hemiparesis, neck pain, and urinary retention	NA	NA	NA	NA	NA
Benndorf(2000)[15]	59/M	6	Neck/R	soft mass in the neck tissue, caused intermittent pain	Yes	Operation /embolization	Recovery	3	NA
Roth(2000)[16]	36/F	0	NA	left shoulder pain	Yes	Operation /embolization	Recovery	6	NA
Hori(2000)[17]	41/F	24	C1 and C3/R	Pulsatile tinnitus	NA	Embolization(Balloon, coil)	Recovery	132	NA
Hori(2000)[17]	51/F	NA	C1/L	Pulsatile tinnitus	NA	Embolization(Polyvinyl alcohol, coil)	Recovery	57.6	NA
Hori(2000)[17]	29/F	NA	C5/L	Pulsatile tinnitus, neck pain	NA	Embolization(Coil and N-Butyl Cyanoacrylate)	Recovery	42	NA
Hori(2000)[17]	66/F	NA	C5/L	Pulsatile tinnitus, neck pain,	NA	Embolization(Coil	Recovery	30	NA

				radiculomyelopathy		and N-Butyl Cyanoacrylate)			
Hori(2000)[17]	51/F	84	C4/R	Pulsatile tinnitus, neck pain, radiculomyelopathy	NA	Embolization(Coil and N-Butyl Cyanoacrylate)	Recovery	38	NA
Kāhārā(2002)[18]	38/M	60	C3-4/R	Neck pain and numbness radiating down to right upper extremity; proximal right upper extremity weakness	NA	Embolization(Coils)	Recovery	24	Trauma
Kubota(2002)[19]	0/M	0	Left posterior fossa	Heart failure; scalp murmur	NA	Embolization(Coils)	Recovery	18	NA
Maheshwari(2002)[20]	30/F	3	C1-6/L and R	Neck pain, progressive paraparesis, deep tendon reflexes were absent, loss of sensation over the extremities, loss of muscle power	NA	Embolization(Coils)	NA	NA	NA
Siddhartha(2003)[21]	36/F	3	C2-3/L and R	neck pain, weakness of all	NA	Embolization(Coils)	Recovery	6	NA

				four limbs					
Hauck(2006)[22]	51/F	NA	C1-7/L	Left rib cage intractable pain, walking difficulty, left hand weakness/numbness	NA	Operation /embolization	Recovery	24	NA
Guzel(2007)[23]	36/M	3	CVJ to the C5/R	progressive weakness of his legs and arms, pain in his left arm, hoarseness	NA	Operation	Neck pain, post laminectomy kyphotic deformity	4	NA
Saito(2007)[24]	62/F	NA	T10-11/L	spastic paraparesis, decrease of sensation in the bilateral lower extremities, and vesicoureteral disturbance	NA	Operation	Recovery	36	NA
Paolini(2008)[25]	26/M	NA	C2-3/NA	sensory disturbances and progressive weakness of 4 limbs	Yes	Operation	minimal weakness of the left hand	12	Trauma
Cohen(2008)[26]	8/F	6	internal carotid artery, ophthalmic artery/L	progressive left pulsatile proptosis	NA	Embolization(Coils)	Recovery	12	NA
Patro(2009)[27]	29/F	3	C3-6/L	humming sound over the left neck, radicular pain in her	NA	Embolization after failed	Recovery	12	NA

				left upper limb, paranesthesia, and progressive weakness of both upper and lower limbs		surgery			
Higa(2010)[28]	60/F	0	NA/L	neck pain and swelling, associated with severe difficulty breathing	Yes	Embolization(Coils)	mild left upper extremity neurologic deficits	3	Operation
Morvan(2011)[29]	36/F	0	C2/L	acute onset of violent and unusual headache, and left cervical neck pain and stiffness	Yes	Embolization(Stenting and coil)	Recovery	NA	NA
Hughes (2012)[30]	29/F	1	C2-7/R	gait difficulty, lower extremity spasticity and neck and arm pain	NA	Embolization(Balloon, coils and glue)	Recovery	17	Trauma
Fukuda(2012)[31]	47/M	0	T7-11/R	sudden back pain	Yes	Embolization(Coils)	Recovery	NA	NA
Gao(2013)[32]	24/F	3	C2-3/L	progressive cervical pain	NA	Embolization(Balloon)	Recovery	6	NA
Gao(2013)[32]	46/F	180	C2/R	progressive neck mass and humming sound over right	NA	Embolization(Balloon, coils	Recovery	6	NA

				neck		and glue)			
Daubner(2015)[33]	46/NA	1.5	C4-6/L	Progressive left neck pain and weakness in the left upper arm	NA	Embolization(Coils)	Recovery	NA	NA
Narayana(2015)[34]	42/M	3	C4-6/R	neck pain, weakness of both the arms, humming sound on the right side of the neck	NA	Embolization(Balloon, coils and glue)	Recovery	12	NA
Uneda(2016)[35]	35/F	0	C3-4/R	severe right neck and shoulder pain	Yes	Embolization(Coils)	Recovery	3	NA
Imahori(2016)[36]	70/F	0	C3-5/L	sudden left neck swelling with pain and bruit	Yes	Embolization(Coils and glue)	Recovery	24	NA
Narayanamurthy(2017)[37]	40/M	NA	Neck/L	previous right MCA infarct, large painful lump on the left neck	NA	Embolization(Coils)	Died	0	NA
He(2018)[38]	31/M	0	superior sagittal, transverse, sigmoid sinuses, confluence of sinuses	sudden onset of loss of consciousness accompanying an episode of focal seizure	NA	NA	NA	NA	NA
Maki(2018)[39]	59/NA	NA	C4/L	comatose state	Yes	Embolization(Coils)	Died of pneumonia	12	NA

Han(2019)[40]	37/M	6	A–V shunting zone at the lateral vein of Galen wall	progressive dizziness and tinnitus	NA	Embolization(Dimethyl sulfoxide)	Recovery	120	NA
Su(2019)[41]	31/F	3	C3-6/L	progressive symptoms presented as numbness and weakness in left side with trouble working and movement disturbance	NA	Embolization	Recovery	NA	NA
Present	39/F	192	C2-5/L	appearing left cervical mass with tremor,	NA	Embolization(Coil)	Recovery	24	NA

Note: M, male; F, female; C, Cervical spine; T, thoracic; L, left side; R, right side; NA, not available.

The following six papers failed to be attained:
Kawasaki(1977)[42], Kamiyama(1985)[43], Anegawa(1997)[44], Sampei(1999)[45], Tanaka(2002)[46] and Takegami(2012)[47].

References:

[1] Deans WR, Bloch S, Leibrock L, et al. Arteriovenous fistula in patients with neurofibromatosis. Radiology1982 Jul;144(1):103-7.
[2] Parkinson D, Hay R. Neurofibromatosis. Surgical neurology1986 Jan;25(1):109-13.
[3] Kubokura T, Sanno N, Koyama S, et al. [Neurofibromatosis with extracranial vertebral arteriovenous fistulae. Case report]. Neurologia medico-chirurgica1987 Dec;27(12):1173-9.
[4] Tsuha M, Kashiwagi S, Katoh S, et al. Traumatic arterio-venous fistula with false aneurysm in neurofibromatosis. Neuroradiology1988;30(1):88.

[5] Hasegawa H, Bitoh S, Katoh A, et al. Bilateral vertebral arteriovenous fistulas and atlantoaxial dislocation associated with neurofibromatosis--case report. Neurologia medico-chirurgica1989 Jan;29(1):55-9.
[6] Wada K, Ohtsuka K, Terayama K, et al. Neurofibromatosis with spinal paralysis due to arteriovenous fistula. Archives of orthopaedic and trauma surgery1989;108(5):322-4.
[7] Schievink WI, Piepgras DG. Cervical vertebral artery aneurysms and arteriovenous fistulae in neurofibromatosis type 1: case reports. Neurosurgery1991 Nov;29(5):760-5.
[8] Terada T, Nakamura Y, Hayashi S, et al. Complex occipital arteriovenous fistula associated with neurofibromatosis treated by embolization and surgery. Case report. Surgical neurology1993 Sep;40(3):245-50.
[9] Cluzel P, Pierot L, Leung A, et al. Vertebral arteriovenous fistulae in neurofibromatosis: report of two cases and review of the literature. Neuroradiology1994 May;36(4):321-5.
[10] Yilmaz M, Ada E, Vayvada H, et al. Management of a large arteriovenous fistula in the face: a case of neurofibromatosis type 1. Annals of plastic surgery1997 Sep;39(3):308-13.
[11] Koenigsberg RA, Aletich V, Debrun G, et al. Cervical vertebral arteriovenous fistula balloon embolization in a patient with neurofibromatosis type 1. Surgical neurology1997 Mar;47(3):265-73.
[12] Murayama Y, Usami S, Abe T, et al. Transvenous Doppler guidewire sonographic monitoring during treatment of a complex vertebral arteriovenous fistula associated with neurofibromatosis type 1. Neuroradiology1999 May;41(5):328-33.
[13] Ushikoshi S, Goto K, Uda K, et al. Vertebral arteriovenous fistula that developed in the same place as a previous ruptured aneurysm: a case report. Surgical neurology1999 Feb;51(2):168-73.
[14] Ma X, Aminina M, Rozen TD. Arteriovenous fistula in neurofibromatosis. Neurology2000 Jul 25;55(2):288.
[15] Benndorf G, Assmann U, Bender A, et al. Vertebral arteriovenous fistula associated with neurofibromatosis type I misdiagnosed as a giant aneurysm. Interventional neuroradiology : journal of peritherapeutic neuroradiology, surgical procedures and related neurosciences2000 Mar 30;6(1):67-74.
[16] Roth TC, Manness WK, Hershey BL, et al. Complex vertebral arteriovenous fistula and ruptured aneurysm in neurofibromatosis: a therapeutically challenging case. Skull base surgery2000;10(1):35-41.
[17] Hori Y, Goto K, Ogata N, et al. Diagnosis and endovascular treatment of vertebral arteriovenous fistulas in neurofibromatosis type 1. Interventional neuroradiology : journal of peritherapeutic neuroradiology, surgical procedures and related neurosciences2000 Sep 30;6(3):239-50.
[18] Kahara V, Lehto U, Ryymin P, et al. Vertebral epidural arteriovenous fistula and radicular pain in neurofibromatosis type I. Acta neurochirurgica2002 May;144(5):493-6.
[19] Kubota T, Nakai H, Tanaka T, et al. A case of intracranial arteriovenous fistula in an infant with neurofibromatosis type 1. Child's nervous system : ChNS : official journal of the International Society for Pediatric Neurosurgery2002 Apr;18(3-4):166-70.
[20] Maheshwari S, Kale HA, Desai SB, et al. Magnetic resonance imaging findings in an unusual case of atlanto axial dislocation and vertebral artery-vein fistulas in a patient of neurofibromatosis-1. Australasian radiology2002 Sep;46(3):316-8.
[21] Siddhartha W, Chavhan GB, Shrivastava M, et al. Endovascular treatment for bilateral

vertebral arteriovenous fistulas in neurofibromatosis 1. Australasian radiology2003 Dec;47(4):457-61.
[22] Hauck EF, Nauta HJ. Spontaneous spinal epidural arteriovenous fistulae in neurofibromatosis type-1. Surgical neurology2006 Aug;66(2):215-21.
[23] Guzel A, Tatli M, Er U, et al. Surgical treatment of cervical arteriovenous fistula in a patient with neurofibromatosis type 1. A case report. The neuroradiology journal2007 Oct 31;20(5):566-9.
[24] Saito A, Takahashi T, Ezura M, et al. Intercostal arteriovenous fistula associated with neurofibromatosis manifesting as congestive myelopathy: case report. Neurosurgery2007 Sep;61(3):E656-7; discussion E57.
[25] Paolini S, Colonnese C, Galasso V, et al. Extradural arteriovenous fistulas involving the vertebral artery in neurofibromatosis Type 1. Journal of neurosurgery Spine2008 Feb;8(2):181-5.
[26] Cohen JE, Gomori JM, Grigoriadis S, et al. Dural arteriovenous fistula of the greater sphenoid wing region in neurofibromatosis type 1. Pediatric neurosurgery2008;44(2):172-5.
[27] Patro SN, Gupta AK, Arvinda HR, et al. Combined transarterial and percutaneous coiling of a spontaneous vertebrovertebral fistula associated with neurofibromatosis Type 1. Case report. Journal of neurosurgery2009 Jul;111(1):37-40.
[28] Higa G, Pacanowski JP, Jr., Jeck DT, et al. Vertebral artery aneurysms and cervical arteriovenous fistulae in patients with neurofibromatosis 1. Vascular2010 May-Jun;18(3):166-77.
[29] Morvan T, de Broucker F, de Broucker T. Subarachnoid hemorrhage in neurofibromatosis type 1: case report of extracranial cerebral aneurysm rupture into a meningocele. Journal of neuroradiology = Journal de neuroradiologie2011 May;38(2):125-8.
[30] Hughes DG, Alleyne CH, Jr. Rare giant traumatic cervical arteriovenous fistula in neurofibromatosis type 1 patient. BMJ case reports2012 Jun 28;2012.
[31] Fukuda W, Taniguchi S, Fukuda Md Phd I. Endovascular treatment of ruptured intercostal arteriovenous fistulas associated with neurofibromatosis type 1. Annals of vascular diseases2012;5(1):109-12.
[32] Gao P, Chen Y, Zhang H, et al. Vertebral arteriovenous fistulae (AVF) in neurofibromatosis type 1: a report of two cases. Turkish neurosurgery2013;23(2):289-93.
[33] Daubner D, Mucha D, Juratli TA, et al. [Neurofibromatosis type 1 associated spinal arteriovenous fistula - treatment with primary endovascular coiling]. RoFo : Fortschritte auf dem Gebiete der Rontgenstrahlen und der Nuklearmedizin2015 Dec;187(12):1130-2.
[34] Narayana RV, Pati R, Dalai S. Endovascular management of spontaneous vertebrovertebral arteriovenous fistula associated with neurofibromatosis 1. The Indian journal of radiology & imaging2015 Jan-Mar;25(1):18-20.
[35] Uneda A, Suzuki K, Okubo S, et al. Neurofibromatosis Type 1-Associated Extracranial Vertebral Artery Aneurysm Complicated by Vertebral Arteriovenous Fistula After Rupture: Case Report and Literature Review. World neurosurgery2016 Dec;96:609.e13-09.e18.
[36] Imahori T, Fujita A, Hosoda K, et al. Endovascular Internal Trapping of Ruptured Occipital Artery Pseudoaneurysm Associated with Occipital-Internal Jugular Vein Fistula in Neurofibromatosis Type 1. Journal of stroke and cerebrovascular diseases : the official journal of National Stroke Association2016 May;25(5):1284-87.
[37] Narayanamurthy H, El-Badawy S, Mukonoweshuro W, et al. A case of spontaneous haematoma from vertebral artery arterio-venous fistula in a patient with neurofibromatosis type

1. British journal of neurosurgery2017 Dec;31(6):736-37.
[38] He D, Li Y, Yu Y, et al. Segmental neurofibromatosis type 1 complicated with multiple intracranial arteriovenous fistulas: A case study. Clinical neurology and neurosurgery2018 May;168:108-11.
[39] Maki Y, Ishibashi R, Fukuda H, et al. Subarachnoid Hemorrhage from Vertebral Arteriovenous Fistula without Perimedullary Drainage: Rare Stroke Hemorrhagic Event in a Patient of Neurofibromatosis Type 1. Neurologia medico-chirurgica2018 Apr 15;58(4):185-88.
[40] Han Z, Du Y, Qi H. Galenic dural arteriovenous fistula in neurofibromatosis type 1 treated with Onyx. Interventional neuroradiology : journal of peritherapeutic neuroradiology, surgical procedures and related neurosciences2019 Dec;25(6):692-96.
[41] Su XJ, Li Q, Shen HX. Arteriovenous Fistula Inside Cervical Spinal Canal Associated with Neurofibromatosis Type 1. World neurosurgery2019 Mar;123:283-85.
[42] Kawasaki M, Miura N, Kagawa M, et al. [A case of spontaneous extracranial vertebral A.V.M. with neurofibromatosis (author's transl)]. No shinkei geka Neurological surgery1977 Jul;5(8):877-82.
[43] Kamiyama K, Endo S, Horie Y, et al. [Neurofibromatosis associated with intra- and extracranial aneurysms and extracranial vertebral arteriovenous fistula]. No shinkei geka Neurological surgery1985 Aug;13(8):875-80.
[44] Anegawa S, Hayashi T, Torigoe R, et al. [Symptomatic arteriovenous fistula in a patient with neurofibromatosis type I]. No shinkei geka Neurological surgery1997 Apr;25(4):373-8.
[45] Sampei T, Yugami H, Sumii T, et al. [A case of neurofibromatosis type 1 associated with arteriovenous fistula caused by re-bleeding of a vertebral dissecting aneurysm]. No shinkei geka Neurological surgery1999 Oct;27(10):927-31.
[46] Tanaka T, Hasegawa Y, Kanki T, et al. [Combination of intravascular surgery and surgical operation for occipital subcutaneous arteriovenous fistula in a patient with neurofibromatosis type I]. No shinkei geka Neurological surgery2002 Mar;30(3):309-13.
[47] Takegami T, Imai K, Umezawa K, et al. [Endovascular trapping using a tandem balloon technique for a spontaneous vertebrovertebral fistula associated with neurofibromatosis type 1]. No shinkei geka Neurological surgery2012 Aug;40(8):705-9.
